# Supplementary material for: Chromothripsis during telomere crisis is independent of NHEJ, and consistent with a replicative origin
Source: Genome Res. 2019 May;29(5):737–49. doi: 10.1101/gr.240705.118 (PMC6499312; doi:10.1101/gr.240705.118)
Supplement: Supplemental Material [file supp_gr.240705.118_Supplemental_file_1.zip › contigs/annotated_contigs/DB114/contig.2.DB114_length_375_mean_cov_4.0.docx]

**DB114_length_375_mean_cov_4.0**

```
CCTCCTCCACATCCACCTCCTCCGCCTCCTCCTCCTCCTCATC|TCTCTCCGCGCACTACACATCCTCCTCCACCCCGCGGCGTCACTC
 >chr7:5352632-5352675 - E=2e-05 p=0e+00
T|TCTCCTCCTCTCCTCCTCTTCCTTCCTCTCCTCCTCTCCTCCTCCTCCTTCCTCTCCTCCTCCACCTCCTCCTTCCTCTCCTCCTCC
  >chr8:49105072-49105358 - E=6e-161
TCCTCTTCCTCCTTATCTTCCCTCTCCTGCTAGGAAGAGCATTAAATAGAACTTGAGATCTTCCCCCTGCTGTACCCAGCAGTTCTTTT

CTAACTGCCTGTGGAACAGTGTTTTGTGTTCGCCCTATTTACCAAAGACCAAAAATTGGTTTGGAGGCAGCTTCTACCTTCTCTTCTAC

CTTTCTAGTTAGTGGTAATTT
```
